# Resection of Left Atrial Myxoma in a Patient with Retrosternal Gastric Tube: a Case Report

**DOI:** 10.21470/1678-9741-2016-0056

**Published:** 2017

**Authors:** Pablo Maria Alberto Pomerantzeff, Elinthon Tavares Veronese, Fabrício José Dinato, Fabio Biscegli Jatene

**Affiliations:** 1 Instituto do Coração do Hospital das Clínicas da Faculdade de Medicina da Universidade de São Paulo (InCor-HCFMUSP), São Paulo, SP, Brazil.

**Keywords:** Heart Neoplasms, Heart Atria, Myxoma, Retrosternal gastric tube

## Abstract

The median sternotomy remains the standard approach in cardiovascular surgery but, in some conditions, it can be considered difficult to perform, especially in patients with history of esophagectomy. This case report describes a successful resection of a left atrial myxoma through a right anterolateral thoracotomy approach in a patient with a previous retrosternal gastric tube reconstruction. The decision for the best surgical approach was made after a heart surgery team discussion. Through this surgical access, a safe and excellent exposure of the left atrium was possible, and a complete resection of the myxoma was performed without any injury to the gastric tube.

**Table:** 

Abbreviations, acronyms & symbols	
CPB	= Cardiopulmonary bypass
MRI	= Magnetic resonance imaging
RGT	= Retrosternal gastric tube

## INTRODUCTION

The median sternotomy remains the standard approach in cardiovascular surgery but, in some conditions, it can be considered difficult to perform, especially in patients with history of esophagectomy. Currently cardiac surgery is still uncommon after an esophagectomy, due to the poor prognosis of esophageal cancer. This case report describes a successful resection of a left atrial myxoma through a right anterolateral thoracotomy approach in a patient with a previous retrosternal gastric tube (RGT) reconstruction for adenocarcinoma of esophagogastric junction.

## CASE REPORT

A 66-year-old male, physician, was admitted to our institute with a left atrial tumor suggesting a myxoma. He had a history of esophagectomy with retrosternal gastric tube reconstruction for adenocarcinoma of gastroesophageal junction, twenty years previously. He was asymptomatic, and the left atrial mass was incidentally discovered by an echocardiogram study during a preoperative evaluation for an ophthalmologic procedure. He had a normal physical examination, without heart murmur or arrhythmia, as well as normal values of laboratory tests.

**Fig. 1:**
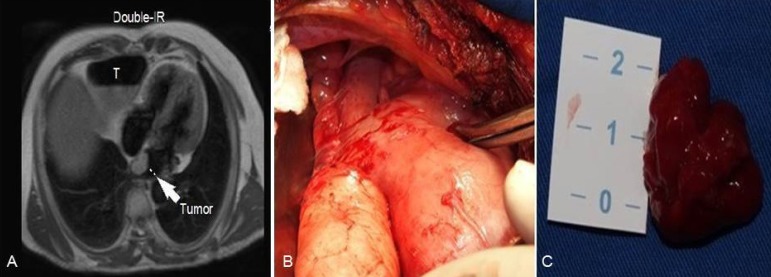
A and B: Relationship among gastric tube (T), heart (tumor) and sternum; magnetic resonance imaging and surgical view. C: Macroscopic aspect of myxoma.

Preoperative transthoracic echocardiography showed normal ventricular and mitral function and a heterogeneous mass in the left atrium attached to the interatrial septum. A heart magnetic resonance imaging (MRI) was performed to establish the anatomic relations among the heart, the gastric tube and the sternum (Figure 1A). The MRI demonstrated an intracardiac lobular mass in the left atrium (2-3 cm in size) attached to the left atrial septum, as well as the relationship between the intrathoracic structures. As it was an unusual case, this imaging study was crucial in order to make the decision for the best surgical approach, which was made after a heart surgery team discussion. The right anterolateral thoracotomy was the approach of choice.

**Fig. 2:**
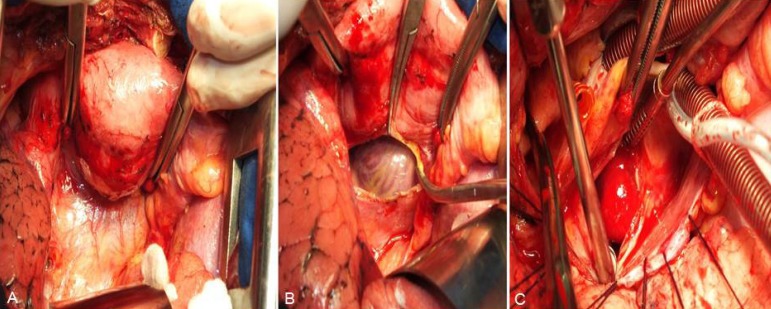
A: Exposure of the right lateral portion of the pericardium after dissection of the gastric tube and the right lung. B: Site of pericardiotomy 20 mm above the phrenic nerve. C: Left atriotomy and visualization of myxoma.

For surgical access, a right anterolateral thoracic incision was made and the pectoralis major muscle was divided. After the pleural space opening through the fourth intercostal space, it was possible to observe the gastric tube adjacent to the posterior sternum portion, as well as many adherences between the lung and the gastric tube (Figure 1B). Through cautious bluntly dissection we have obtained a wide view of the lateral portion of the pericardium (Figure 2A). About 20 mm anterior to the right phrenic nerve, a longitudinal pericardium incision was made and it allowed an adequate exposure of the heart and the great vessels (Figure 2B).

Moderate hypothermic (28ºC) cardiopulmonary bypass (CPB) was established with left femoral artery cannulation and venous cannulas placed in the superior and inferior vena cava. The aortic cross-clamping was performed in a standard manner. After the administration of cardioplegic solution in the aortic root, a longitudinal left atriotomy was performed with a good exposure of the tumor, which had a macroscopic aspect of an intracardiac myxoma (Figure 2C). It was possible to resect the entire tumor without any injury to the interatrial septum (Figure 1C). The patient was easily weaned from CPB and the incision was closed. The CPB and the arterial cross-clamp times were 40 minutes and 26 minutes, respectively.

The patient had an uneventful postoperative course, with a length of stay in the intensive care unit of 36 hours. The predischarge transthoracic echocardiography demonstrated a normal biventricular function, with a complete resection of the tumor and an intact interatrial septum. The patient was discharged to home on postoperative day 5, and the histopathologic exam confirmed the diagnosis of myxoma. After nineteen months of follow-up, medical surveillance shows no complication (Table 1).

**Table 1:** Data from this case report organized in chronological order.

**Dates**	**Past Medical History**		
Twenty years before surgery	History of esophagectomy for gastroesophageal junction carcinoma reconstructed with a retrosternal gastric tube		
**Dates**	**Initial Evaluation and Follow-up**	**Diagnostic Testing**	**Interventions**
Five months before surgery	Asymptomatic, being submitted to a preoperative evaluation for an ophthalmologic procedure	Echocardiogram study incidentally discovered a left atrial mass	Referred to cardiologist and cardiac surgeon
Three months before surgery	Heart surgery team discussion	Heart magnetic resonance imaging was performed to establish the anatomic relations among the heart, the gastric tube and the sternum	The right anterolateral thoracotomy was the approach of choice
Day of surgery	Day of surgery		Resection of the intracardiac tumor without injury to the gastric tube
Fifth postoperative day	Discharge from hospital uneventfully	Echocardiographic study demonstrated complete resection of the tumor and an intact interatrial septum	
Nineteen months after surgery	Asymptomatic, normal physical examination		To continue medical follow-up

## DISCUSSION

Despite the advances in the management of esophageal cancer, a cardiac surgery - after an esophagectomy with RGT reconstruction - is still rare, and the prognosis of this entity justifies this condition^[1,2]^. In these patients, the presence of a RGT raise concerns about the ideal surgical approach to expose the heart without any injury to the digestive conduct, which could result in severe sepsis.

Although median sternotomy remains the conventional approach in cardiovascular surgery, alternate incisions for reoperations must be selected to avoid an injury to the reconstructed gastric tube and the surrounding tissues^[3]^.

To the best of our knowledge, there are 14 case reports about cardiovascular surgery in patients with RGT, all related to aorta or valvular surgeries. Our case is the first about the resection of a left atrial myxoma in a patient with RGT reconstruction. The approaches reported were median sternotomy, right parasternal incision and left thoracotomy^[4]^.

The use of left thoracotomy has been previously reported and recommended for aortic valve replacement after an esophagectomy with RGT reconstruction^[5]^, just like the median sternotomy has been considered a feasible approach of the aortic valve after a careful examination of the gastric tube and the gastroepiploic artery^[4]^. Gillinov et al.^[2]^ successfully chose the right parasternal incision for aortic valve replacement in a case with history of substernal colon interposition. Also, Mazzitelli et al.^[6]^ described a right parasternal approach for aortic valve replacement, after a retrosternal gastropexy.

Preoperative imaging exams are essential to decide for the better surgical access. This decision may be determined by the location of the gastric tube as well as the position of the gastroepiploic artery, which is responsible for the blood supply of the digestive conduct^[2,3]^. Computed tomography is the most used imaging study in these cases, probably due to its availability. However, MRI also may be used in preoperative evaluation, as described in this case.

Since all surgical approaches have advantages and disadvantages, we highlight the importance of discussion and individualization of the surgical strategy for each situation.

Being an unusual case, the discussion about the best and safer surgical approach with other surgeons was crucial and it promoted the success of the procedure. In the present case, previous experience and the characteristics of the imaging study determined the decision.

With a right anterolateral thoracotomy, it was possible a safe dissection and retraction of the RGT, assuring excellent exposition of heart and great vessels. The femoral artery cannulation also contributed to this good surgical view.

In conclusion, it is possible to say that a right anterolateral thoracotomy is an applicable and safe approach for treatment of left atrial myxoma in patients with RGT reconstruction.
